# Integrated analysis of the involvement of nitric oxide synthesis in mitochondrial proliferation, mitochondrial deficiency and apoptosis in skeletal muscle fibres

**DOI:** 10.1038/srep20780

**Published:** 2016-02-09

**Authors:** Gabriela Silva Rodrigues, Rosely Oliveira Godinho, Beatriz Hitomi Kiyomoto, Juliana Gamba, Acary Souza Bulle Oliveira, Beny Schmidt, Célia Harumi Tengan

**Affiliations:** 1 Department of Neurology & Neurosurgery, Escola Paulista de Medicina, Universidade Federal de São Paulo;São Paulo, Brazil; 2 Division of Cellular Pharmacology, Escola Paulista de Medicina, Universidade Federal de São Paulo, São Paulo, Brazil; 3 Department of Pathology, Escola Paulista de Medicina, Universidade Federal de São Paulo, São Paulo, Brazil

## Abstract

Nitric oxide (NO) is an important signaling messenger involved in different mitochondrial processes but only few studies explored the participation of NO in mitochondrial abnormalities found in patients with genetic mitochondrial deficiencies. In this study we verified whether NO synthase (NOS) activity was altered in different types of mitochondrial abnormalities and whether changes in mitochondrial function and NOS activity could be associated with the induction of apoptosis. We performed a quantitative and integrated analysis of NOS activity in individual muscle fibres of patients with mitochondrial diseases, considering mitochondrial function (cytochrome-*c*-oxidase activity), mitochondrial content, mitochondrial DNA mutation and presence of apoptotic nuclei. Our results indicated that sarcolemmal NOS activity was increased in muscle fibres with mitochondrial proliferation, supporting the relevance of neuronal NOS in the mitochondrial biogenesis process. Sarcoplasmic NOS activity was reduced in cytochrome-c-oxidase deficient fibres, probably as a consequence of the involvement of NO in the regulation of the respiratory chain. Alterations in NOS activity or mitochondrial abnormalities were not predisposing factors to apoptotic nuclei. Taken together, our results show that NO can be considered a potential molecular target for strategies to increase mitochondrial content and indicate that this approach may not be associated with increased apoptotic events.

Nitric oxide (NO) has gained much attention in the past decades in several research fields due to its properties as a free radical and signaling molecule in different cellular pathways. Its relevance in cellular physiology is supported by the fact that NO is synthesized in several tissues by the enzymes NO synthases (NOS). These enzymes generate NO by catalyzing the conversion of L-arginine to L-citrulline and are found as three isoforms: neuronal (nNOS), endothelial (eNOS) and inducible (iNOS) NOS^1^. The neuronal and endothelial isoforms are constitutive and regardless of the original designations, are also expressed in other tissues including skeletal muscle^2^; while iNOS is only expressed under certain circumstances, such as during defense responses against infections or inflammation^3^. Interestingly, in human skeletal muscle nNOS is confined to the sarcolemma (muscle cell membrane), linked to the dystrophin-glycoprotein complex^2^, while eNOS is localized in the inter-myofibrilar region (sarcoplasm)^4^,^5^, suggesting that each isoform may have different roles in muscle physiology.

NO has important participation in different aspects of repair mechanisms of the skeletal muscle^6^. After muscle injury, the myogenic precursors (satellite cells) are activated and proliferate to regenerate the muscle cells. NO stimulates the proliferation and activation of satellite cells, maintaining the regenerative capacity of adult skeletal muscle^7^. During myogenesis, NO inhibits the dynamin related protein 1 (DRP1) activity, promoting mitochondrial elongation, which is important for the formation of the mitochondrial network and myogenic differentiation^8^. Furthermore, Cordani *et al.*^9^ have demonstrated that NO can also induce the inhibition of fibrosis and adipogenesis in skeletal muscle. Thus NO is considered a promising target for treatment of muscle diseases with the aim to induce regeneration, reduce fat infiltration and inhibit fibrosis. However, the exact mechanisms of NO-based pharmacological approaches are probably complex and involve not only the direct effects as a signaling molecule, but also post-translation modifications and epigenetic regulation. In fact, studies with dystrophic muscles show that the histone deacetylase 2 (HDAC2) is up-regulated and the treatment with an NO-donor promotes the inhibition of this enzyme by S-nitrosylation^10^, demonstrating that NO may affect gene expression by epigenetic mechanisms.

Besides the action in the repair mechanisms, NO is also an important signaling molecule for several mitochondrial pathways. NO can act as a modulator of the respiratory chain mainly by inhibiting cytochrome-*c*-oxidase (COX, Complex IV) and NADH:ubiquinone-oxidoreductase (Complex I). At the same time, these inhibitions can lead to superoxide release, predisposing to oxidative stress. Conversely, when participating in redox reactions, NO can also regulate intra-mitochondrial concentration of reactive species, such as NO itself, superoxide radical and peroxynitrite^11^. Likewise, NO can induce pro-apoptotic signals through the opening of mitochondrial permeability transition pore^12^ or by inhibiting apoptosis when caspase-3 is inactivated via S-nitrosation^13^. In another pathway, NO is also involved in the activation of the peroxisome proliferator-activated receptor-γ co-activator 1α (PGC-1α), which is the final step of the mitochondrial biogenesis process^14^,^15^,^16^,^17^,^18^,^19^,^20^. Interestingly, the result of activation of mitochondrial biogenesis is frequently found in muscle biopsies from patients with mitochondrial diseases, which display muscle fibres with intense mitochondrial proliferation, the so-called ragged red fibres (RRF)^21^,^22^. The presence of RRF and the easy detection of mitochondrial deficiencies by muscle histochemistry are interesting features that allow the investigation of several mitochondrial processes in patients with mitochondrial diseases.

These diseases are caused by mutations in nuclear or mitochondrial DNA (mtDNA) encoded genes and have extremely variable phenotypes, ranging from pure myopathies to complex multisystem syndromes in all age range^21^,^23^. When skeletal muscle is affected, COX deficiency can be found in scattered fibres with or without mitochondrial proliferation, pattern that is usually associated with an mtDNA defect^24^. Diffuse mitochondrial deficiencies of Complex IV and II are also found by histochemistry of muscle from patients with nuclear gene mutations^24^,^25^. Since the early descriptions of muscle biopsy findings in mitochondrial myopathies, mitochondrial proliferation has been considered a compensatory mechanism to improve cellular energetic capacity. Based on this idea, the stimulation of mitochondrial biogenesis has also been considered as a therapeutic strategy to improve mitochondrial deficiencies^14^,^26^,^27^. However, it is still unknown whether the excessive induction of new mitochondria could lead to adverse effects such as increasing the level of mutated mtDNA, oxidative damage or inducing apoptotic events. Although it has been hypothesized that RRF could be associated with apoptosis, the results from several studies were contradictory, which makes this point still controversial^28^,^29^,^30^,^31^,^32^,^33^,^34^. Furthermore, the relevance of NO in the muscle fibre with a respiratory chain deficiency and/or mitochondrial proliferation is still not elucidated. A few studies, including one from our group, have demonstrated increased nNOS expression and reduced NOS activity in RRF in patients with mitochondrial diseases^4^,^35^. However, these studies were limmited by the small number of patients and the fact that the sarcolemmal NOS activity was determined by visual evaluation, which could predispose to errors or lack of sensitivity.

Considering the involvement of NO in mitochondrial biogenesis, respiratory chain control and apoptotic pathways, we aimed to verify the influence of different mitochondrial abnormalities on NOS activity and whether mitochondrial abnormalities or alterations in NOS activity could be associated with the induction of apoptosis. To answer these questions we performed a combined analysis of an *in situ* quantification of NOS activity, presence of mitochondrial abnormalities and apoptotic nuclei in single muscle fibres. Our main results showed that sarcolemmal and sarcoplasmic NOS activities were altered in muscle fibres with mitochondrial abnormalities such as mitochondrial proliferation and reduction of COX activity, but were not affected by defects of other respiratory chain complexes, such as complex I or II. Additionally, alterations in NOS activity or presence of mitochondrial abnormalities did not predispose to increased apoptotic nuclei in skeletal muscle fibres.

## Results

### Classification of muscle fibres according to succinate dehydrogenase (SDH) and COX staining

**Figure 1:**
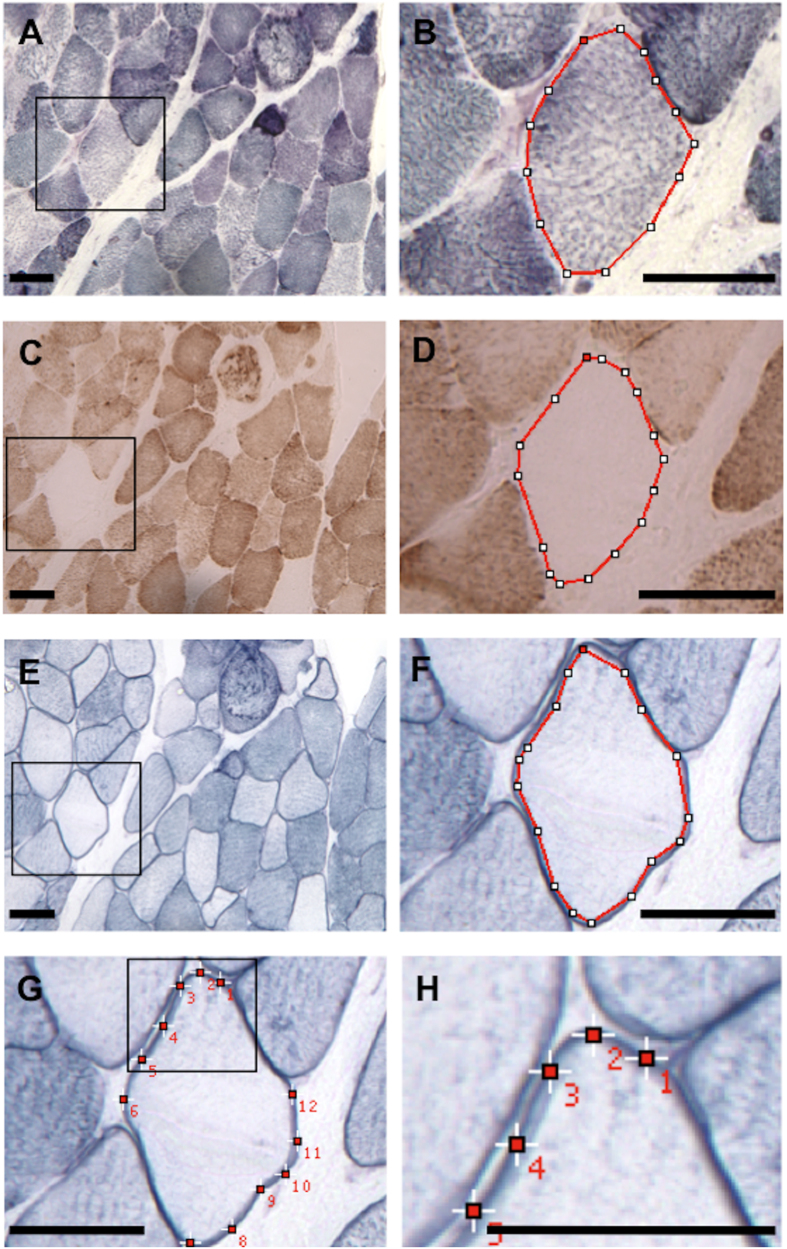
Histochemical quantification in skeletal muscle. Muscle section from a patient with SDH (**A**,**B**), COX (**C**,**D**) and NADPHd (**E–H**) histochemistry. We show an example of a COX− fibre with reduced sarcoplasmic NOS and increased sarcolemmal NOS activities. SDH (**B**), COX (**D**) and sarcoplasmic NOS (**E**,**F**) activities were measured by circumscribing (red line) the sarcoplasm, with an interactive cursor, excluding the sarcolemmal membrane in the case of sarcoplasmic NOS activity. Sarcolemmal NOS activity (**G**,**H**) was obtained by placing fixed size squares at 12 different sites on the sarcolemmal membrane. Care was taken to avoid areas with superimposed membranes. The mean optical density (O.D) measured inside the squares and circumscribed areas was considered as an estimate of the histochemical activity. Panels (**B**,**D**,**F**,**H**) are amplified images delimitated by the black square frame on (**A**,**C**,**E**,**G**), respectively. SDH: succinate dehydrogenase, COX: cytochrome-*c*-oxidase, NADPHd: NADPH diaphorase, COX− = COX negative. Scale bar = 50 μm.

**Figure 2:**
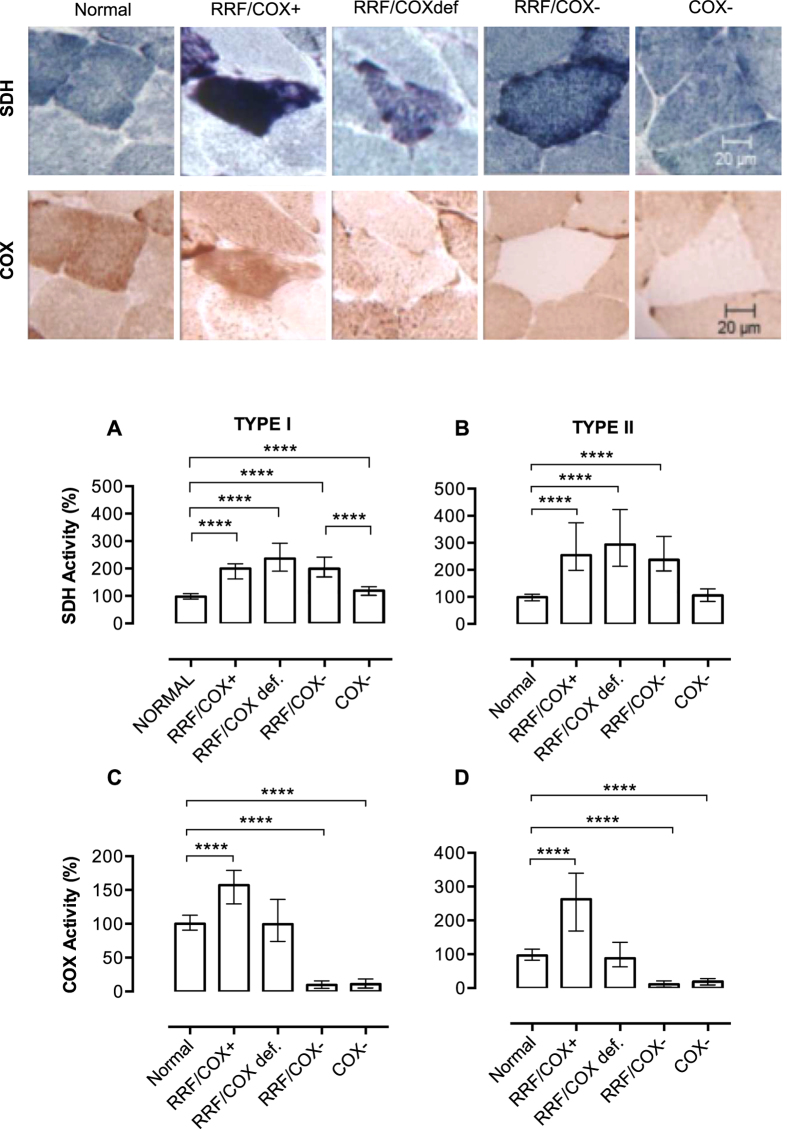
Types of abnormalities in muscle fibres after SDH and COX histochemistry. The panel on top shows examples of the different types of muscle fibres included in this study on SDH and COX histochemistry. From the left to the right, we demonstrate an example of normal muscle and each type of abnormal fibres: fibre with mitochondrial proliferation and preserved COX activity (RRF/COX+), fibre with mitochondrial proliferation and impaired COX activity (RRF/COXdef), fibre with mitochondrial proliferation and low COX activity (RRF/COX−), fibre with low COX activity and without mitochondrial proliferation (COX−). SDH quantification shows that all fibres with mitochondrial proliferation (RRF/COX+, RRF/COXdef, RRF/COX−) have higher values when compared to normal fibres (**A**,**B**). Type I COX− fibres have also increased SDH staining, but is significantly different when compared to RRF/COX− fibres showing that it is a distinct group. COX quantification (**C**,**D**) demonstrates that RRF/COX+ fibres have increased COX activity while RRF/COX− and COX− fibres had lower activities. The total numbers of myofibres analysed in each group are shown in Table 2. Data were analysed by Kruskal-Wallis test followed by Dunn’s post hoc test. ****P ≤ 0.0001. Bars are showing median and interquartile range.

Muscle biopsies from patients with mitochondrial diseases usually display different degrees and combinations of mitochondrial alterations, which include increase in mitochondrial content and decreased COX activity. To better classify these abnormalities we performed a quantification of SDH and COX histochemical stainings in single muscle fibres as described in Methods and Fig. 1. SDH (Complex II of the mitochondrial respiratory chain) is used to evaluate mitochondrial content, because it is preserved in patients with mtDNA mutations. All the analyses were performed in two groups of fibres, type I (slow-twitch) and II (fast-twitch) fibres, due to differences in oxidative capacity. We evaluated 842 normal (type I, n = 477; type II, n = 365) and 1135 abnormal fibres (type I n = 657, type II n = 478) in muscle biopsies of 24 patients (Table 1). Abnormal fibres were further classified according to the presence of mitochondrial proliferation and COX deficiency (Table 2) in: RRF/COX+ (with mitochondrial proliferation and COX activity similar to normal fibres), RRF/COXdef (with mitochondrial proliferation and disproportional low COX activity), RRF/COX− (with mitochondrial proliferation and low COX activity) or COX− (with low COX activity but no mitochondrial proliferation). Suitability of our classification criteria was confirmed as all groups identified as having mitochondrial proliferation (RRF/COX+; RRF/COX def, RRF/COX−) presented increased levels of SDH activity (P < 0.0001; Fig. 2A,B) and those with impaired COX activity (RRF/COX−, COX−) had significant lower COX staining (Fig. 2C,D) as compared to normal fibres.

It is noteworthy that although type I COX− fibres had a slight increase in SDH activity (median = 119.5%, P < 0.0001) when compared to normal fibres, this increment was not as high as those observed in fibres classified as RRF (RRF/COX+ median = 199.6%; RRF/COXdef median = 236.1%; RRF/COX− median = 199.2%). Type I COX− fibres had SDH activity within the normal range but distributed towards higher values, which explains the higher median (Supplementary Fig. S1). Furthermore SDH staining in type I COX− fibres was significantly lower than in RRF/COX− fibres (P < 0.0001), showing that type I COX− fibres constituted a distinct group (Fig. 2A).

### NADPH diaphorase (NADPHd) activity and mitochondrial abnormalities

**Figure 3:**
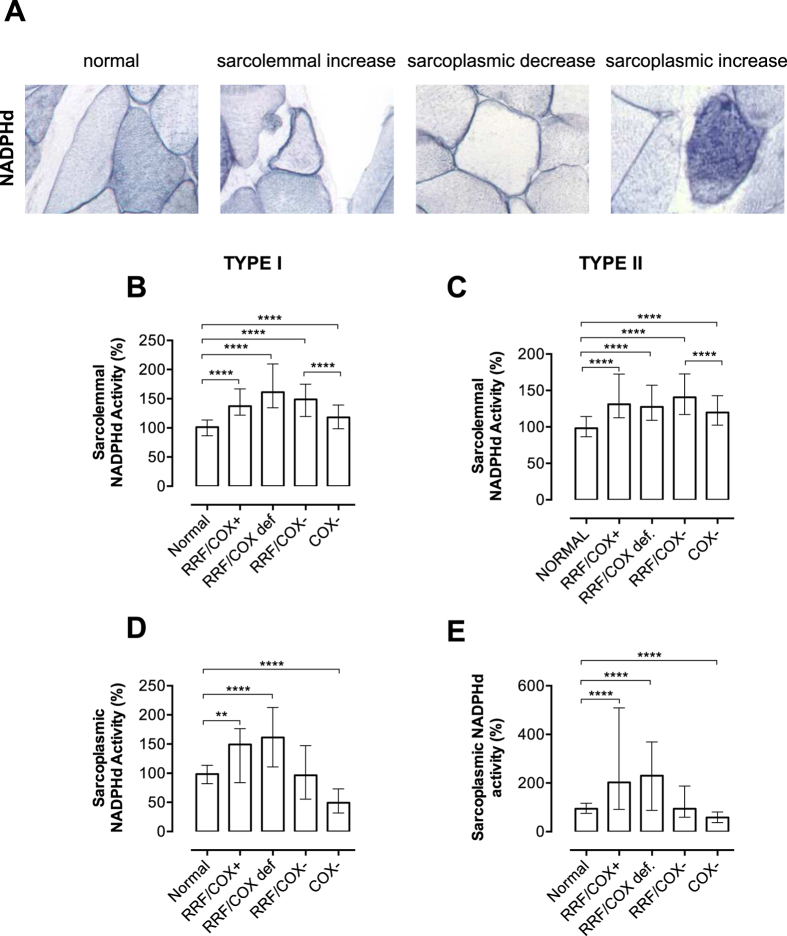
Quantification of NADPHd activity. Sarcolemmal and sarcoplasmic NADPHd stainings are shown in normal and abnormal fibres (**A**). Note that normal fibres have a sarcolemmal staining and type I fibres have darker sarcoplasmic staining. We demonstrate the three types of alterations in NADPHd activities: sarcolemmal increase, sarcoplasmic decrease and sarcoplasmic increase. Quantification of NADPHd activity considering the different types of mitochondrial abnormalities show that sarcolemmal activity is increased in all groups with mitochondrial abnormalities (mitochondrial proliferation or COX deficiency, **B**,**C**). Sarcoplasmic activity is increased in RRF/COX+ and RRF/COXdef fibres, and reduced in COX− fibres when compared to normal fibres. The total numbers of myofibres analysed in each group are shown in Table 2. Data were analyzed by Kruskal-Wallis test followed by Dunn’s post hoc test: ****P ≤ 0.0001, **P ≤ 0.001. Bars are showing median and interquartile range.

The quantification of NADPHd histochemistry was used to evaluate NOS activity in the sarcolemma and sarcoplasm and allowed us to detect NADPHd alterations in fibres with mitochondrial abnormalities (Fig. 3A). Sarcolemmal NADPHd was increased in fibres with mitochondrial proliferation or COX deficiency with medians ranging from 118.0% to 161.3% (Fig. 3B,C). Interestingly, the comparison of the two groups of fibres with low COX activity (RRF/COX− vs. COX−) showed that sarcolemmal NADPHd activity was higher in the group with mitochondrial proliferation (RRF/COX−: type I = 148.8%; type II = 140.7% vs. COX−: type I = 118.0%; type II = 119.8%). This result added to the fact that the groups of fibres with mitochondrial proliferation had higher sarcolemmal NADPHd activities, suggest that mitochondrial proliferation may be an important factor in the up-regulation of this enzyme.

The analysis of sarcoplasmic NADPHd suggested that NADPHd was related to COX activity as NADPHd activity was reduced in COX− fibres (type I = 49.3%; type II =  58.3%; Fig. 3D,E) and increased in fibres with mitochondrial proliferation and positive COX staining (RRF/COX+: type I = 149.2%; type II = 202.2%; RRF/COXdef: type I = 161.0%; type II = 230.3%).

**Figure 4:**
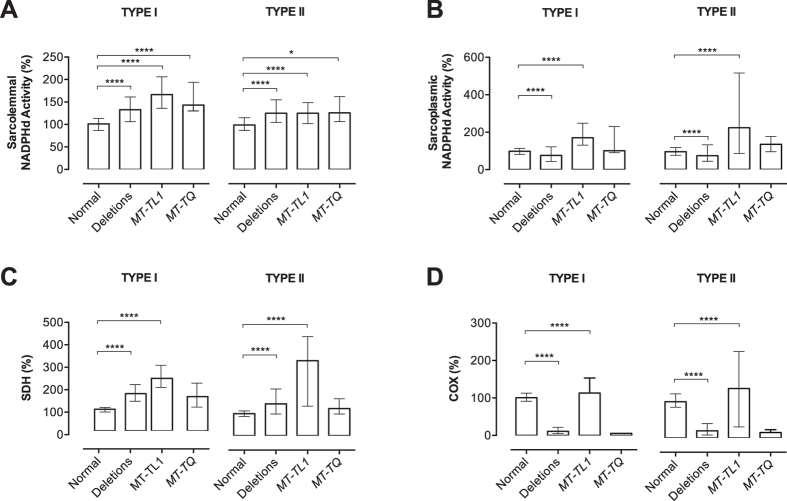
NADPHd activity and genotype in individual muscle fibres. Quantification of NADPHd staining show increased sarcolemmal activity in both types I and II abnormal fibres, regardless of the kind of mutation (**A**). Sarcoplasmic NADPHd activity is also significant increased in patients with *MT-TL1* mutations but decreased in those with mtDNA deletions (**B**). A similar pattern is shown when we analyze SDH (**C**) and COX (**D**) staining in the same groups of fibres classified according to the genotype. SDH activity is increased in fibres with mtDNA deletions and *MT-TL1* mutations. COX activity is increased in *MT-TL1* mutations but reduced in mtDNA deletions. Total numbers of myofibres analysed: deletions (type I: n = 484; type II; n = 392); *MT-TL1* (type I: n = 141; type II: n = 72); *MT-Q* (type I: n = 9; type II: n = 6) and normal (type I: n = 418; type II: n = 332). Data were analyzed by Kruskal-Wallis test followed by Dunn’s post hoc test. ****P ≤ 0.0001; *P ≤ 0.05. Bars are showing median and interquartile range.

Since the groups of abnormal fibres were from patients with different mtDNA mutations, such as mtDNA deletions or mutations in the tRNA^Leu(UUR)^ (*MT-TL1*) and tRNA^Gln^ (*MT-TQ*) genes, we also verified whether NADPHd activity was affected by the mitochondrial genotype. Considering that the effects of a particular mutation are more prominent in abnormal fibres as they contain high levels of mutated mtDNA, we re-classified the abnormal fibres in three groups according to the genotype: mtDNA deletions, *MT-TL1* or *MT-TQ* mutations. With this approach, we still found increased sarcolemmal NADPHd activity in all groups of abnormal fibres (Fig. 4A) with median values ranging from 124.3% (type II fibres with deletions and *MT-TL1* mutation) to 166.6% (type I fibres with *MT-TL1* mutation). On the other hand, sarcoplasmic NADPHd activity was decreased in fibres with mtDNA deletions (type I = 75.4%; type II = 72.8%; vs. normal fibres: type I = 98.6%; type II = 94.1%) and increased in fibres with the *MT-TL1* mutations (type I = 170.4%; type II = 221.1%; Fig. 4B). The analysis of fibers with the *MT-TQ* mutation did not reveal any statistical significant difference in sarcoplasmic NADPHd

Because COX activity is affected in different ways in muscle fibres with mtDNA deletions and *MT-TL1* mutations, NADPHd alterations could be due to differences in SDH or COX activities. For this reason, we analyzed SDH and COX staining in the same groups of fibres classified by genotype. We found that SDH staining was increased in fibers with deletions and with *MT-TL1* mutations (Fig. 4A,C). The analysis of COX activity showed a similar pattern as sarcoplasmic NADPHd, with decrement in abnormal fibers with mtDNA deletions and increment in fibers with the *MT-TL1* mutation (Fig. 4B,D). These results indicate that changes in NADPHd activity correlate with variations of SDH and COX activity rather than the mtDNA mutation.

**Figure 5:**
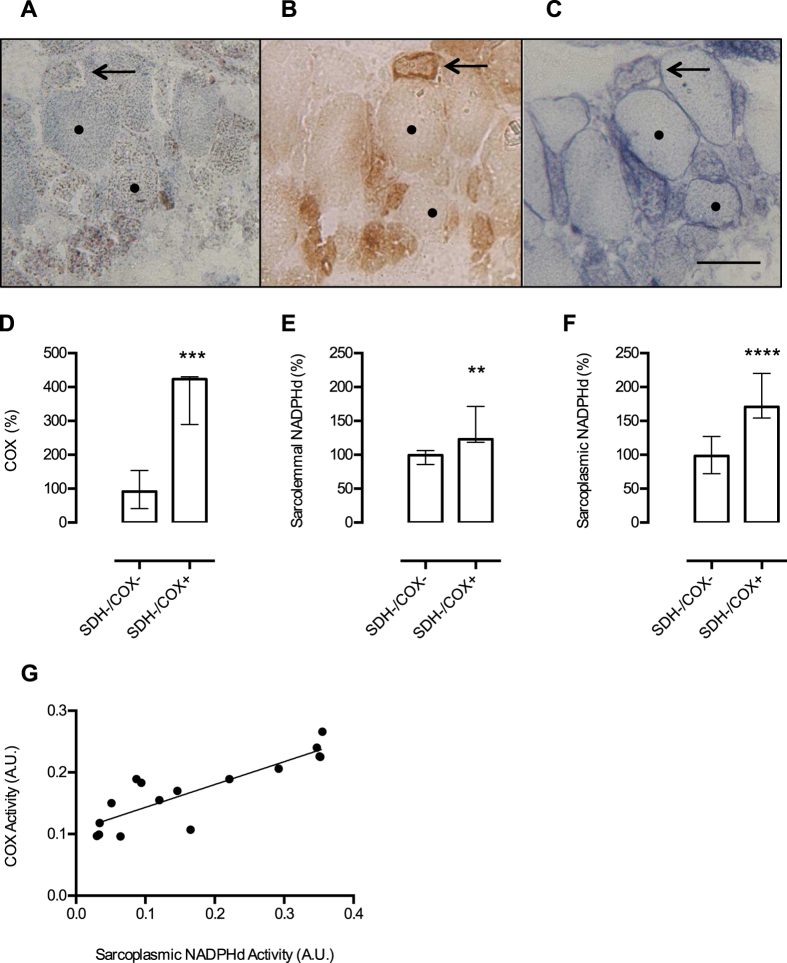
NADPHd activity in muscle fibres and Complex II deficiency. Muscle biopsy from a patient with a complex pattern of mitochondrial enzyme deficiency (complex I, II and IV deficiencies) shows a diffuse lack of SDH staining (**A**). COX histochemistry (**B**) shows fibres with low (•) and increased (arrow) COX activities, showing that mitochondrial content was increased (RRF). NADPHd (**C**) shows that despite SDH deficiency, the same pattern observed in other patients is present: low sarcoplasmic NOS activity in COX− fibre (•) and increased sarcolemmal and sarcoplasmic NOS activities in RRF/COX+ fibre (arrow). Based on COX activity, we separated the fibres in two groups: SDH-/COX− (n = 10) and SDH-/COX+ (n = 8). After quantification of COX staining, we show that the group of SDH-/COX+ fibres has significantly increased COX activity (P = 0.0005; D). NADPHd activities were increased in both sarcolemmal (P = 0.0014; E) and sarcoplasm (P < 0.0001; F) in SDH-/COX+ muscle fibres. There is a good correlation between COX and sarcoplasmic NADPHd activities (n = 16; r = 0.86; G). A.U. = arbitrary units. Data presented in D, E, and F were analyzed by Mann-Whitney test, ** P ≤ 0.01, ***P ≤ 0.001, ****P ≤ 0.0001. Scale bar = 50 μm. Bars are showing median and interquartile range.

We also analyzed two additional patients with different patterns of mitochondrial deficiency and distinct etiology (Patients 30 and 31, Table 1). They could not be included in the previous quantitative analysis due to the absence of normal fibres (patient 30) or normal pattern of SDH and COX staining (patient 31). Muscle biopsy of patient 30 showed a diffuse complex II deficiency, RRF/COX+ fibres, scattered COX− fibres and a Complex I deficiency, as reported previously^36^. Although the genetic defect is unknown, the unique feature of diffuse SDH deficiency allowed us to verify whether SDH activity could have any effect on NADPHd activity. Despite the lack of SDH activity in all fibres, we clearly identified a similar pattern of NADPHd activity as seen in the other patients (Fig. 5A–C). Due to SDH and COX deficiency, we were not able to precisely identify fibres containing mitochondrial proliferation. However, we distinguished two groups of fibres: (1) with low COX and SDH activities (SDH-/COX−) and (2) with low SDH and positive COX activity (SDH-/COX+; Fig. 5D). Sarcolemmal NADPHd activity was increased in SDH-/COX+ fibres (Fig. 5E) suggesting that Complex II deficiency had no influence on NOS activity. Sarcoplasmic NADPHd was higher in SDH-/COX+ than in SDH-/COX− fibres (P < 0.0001; Fig. 5F). This pattern was similar to that found in the other patients, indicating a relationship between the sarcoplasmic NADPHd and COX activity. Because this patient had fibres with different levels of COX activity, it was possible to show a positive correlation between sarcoplasmic NADPHd and COX activity (r = 0.86; Fig. 5G).

At this point, we verified the effects of abnormalities in Complex II and IV on NADPHd activity. To evaluate the impact of a Complex I defect we also analyzed a muscle sample (patient 31) with a mutation in a Complex I subunit gene (NADH dehydrogenase 6). This mutation was homoplasmic and resulted in Complex I deficiency with normal SDH and COX activities on muscle biopsy^37^. No abnormality was found on NADPHd activity (Supplementary Fig. S2), suggesting that, at least in this case, the Complex I defect had little or no effect on NOS activity. Unfortunately, no additional material was available for biochemical studies.

### Apoptosis and NOS activity

Using apoptotic nuclei, detected by the terminal transferase dUTP nick end labeling (TUNEL) assay, as a marker of apoptosis, we also evaluated whether mitochondrial abnormalities or alterations in NADPHd activity could be associated with the induction of apoptosis. We found apoptotic nuclei in 81% of patient samples (21/26) but at low levels, ranging from 0.02% to 1.79% (mean  = 0.47%) of fibre nuclei. No apoptotic nuclei were found in muscle from four patients with mtDNA mutations (patients 14, 28, 29 and 31 in Table 1) and in five control muscles. The presence and proportion of apoptotic nuclei were not affected by age, duration of disease, phenotype or proportion of RRF/COX deficient fibres. If we consider the total number of muscle fibres (n = 1980) analyzed from all patients, only 1.9% had apoptotic nuclei. We also evaluated the possible correlation between mitochondrial/ NADPHd alterations and apoptosis analyzing group of fibres with (TUNEL+; n = 38) and without (TUNEL−; n = 829) apoptotic nuclei (Table 3). The proportions of fibres with mitochondrial abnormalities were similar in TUNEL+ (36.8%) and TUNEL− (43.2%) fibres and in both groups, sarcoplasmic and sarcolemmal NADPHd alterations appeared in similar proportions. These results showed that the presence of mitochondrial abnormalities or alterations in NADPHd activity was not a determinant factor in the appearance of apoptotic nuclei.

## Discussion

Several studies have demonstrated the involvement of NO in mitochondrial signaling pathways^15^,^16^,^17^,^18^,^19^,^38^, but only a few focused on the roles of NO in skeletal muscle with mitochondrial diseases^4^,^35^. One possible reason to explain this paucity of studies is that the direct measurement of NOS activity in muscle homogenates is not appropriate in these cases because fibres with mitochondrial abnormalities usually have a scattered distribution throughout the muscle section. Furthermore, it is not possible to determine the activities of the different NOS isoforms due to their specific locations within the muscle cell. NADPHd histochemistry has been used as an indirect method to detect NOS^2^,^35^,^39^,^40^,^41^,^42^, based on the fact that all NOS isoforms have NADPHd activity^42^ and that there is a good correlation between NADPHd activity and NOS expression in skeletal muscle^4^,^43^. Taking advantage of a quantitative histochemical method for localized NADPHd activity, we were able to measure the NADPHd staining in different muscle fibre compartments and performed an integrated evaluation of NADPHd activity, mitochondrial function and presence of apoptosis. Thus we manage to make a precise classification of mitochondrial abnormalities, which is usually based on qualitative assessment leading to inaccuracy in the discrimination of intermediate states of COX and SDH activities. Furthermore, the quantitative analyses used in the present study allowed us to have a more accurate evaluation of sarcoplasmic and sarcolemmal NADPHd activities in a single fibre.

Our results clearly showed that sarcolemmal NADPHd activity was increased in the three genotypes studied (mtDNA deletions, mutations in the *MT-TL1* and *MT-TQ*) while sarcoplasmic NADPHd activity was low in mtDNA deletions and high in *MT-TL1* mutations. However, a more detailed analysis revealed that, in fact, these changes were closely related to SDH and COX activities, rather than the genotype. Different patterns of SDH and COX staining can be found in different mtDNA mutations. Patients with mtDNA deletions have predominantly COX deficient fibres while those with the m.3243A > G (*MT-TL1* mutation) and MELAS (mitochondrial encephalomyopathy with lactic acidosis and stroke like episodes) phenotype, usually have preserved COX activity. In this particular phenotype, the mutation loads in RRFs do not reach the threshold for COX deficiency, which explains the preservation of COX activity^44^. Interestingly, sarcoplasmic NADPHd activity was decreased in fibres with COX deficiency and there was a positive correlation between COX and sarcoplasmic NADPHd. As we have previously hypothesized^4^, considering that NO promotes an inhibition of Complex IV and that the expression of NOS in the sarcoplasm is preserved, it is possible that NOS becomes down regulated in response to COX deficiency as an attempt to preserve COX function. The higher sarcoplasmic NADPHd activity in patients with *MT-TL1* mutations can be explained by the fact that COX activity is not severely affected in this group. It is still unclear which NOS isoform is responsible for this sarcoplasmic activity, but the increased sarcoplasmic NADPHd in RRF/COX+ fibres supports the existence of an NOS isoform localized in mitochondria or linked to mitochondrial membrane. Some studies have demonstrated the expression of eNOS in the sarcoplasm and in close association with mitochondria^4^,^45^.

The finding that NADPHd activity can be high in the sarcolemma and low in the sarcoplasm in the same muscle fibre supports the idea that NO may have different functions depending on its source. Considering that NO is a Complex IV inhibitor, sarcoplasmic NO production may be necessary for the regulation of the respiratory chain; while NO produced in the sarcolemma could be involved in mitochondrial biogenesis. Based on the location of NADPHd activity, we speculate that eNOS cannot be the source of NO involved in mitochondrial proliferation because RRF had decreased sarcoplasmic NOS activity. It is more plausible to think that nNOS would be responsible for this signaling, which is supported by the finding of a positive correlation between nNOS and a mitochondrial marker in muscle from patients with mitochondrial diseases^4^.

The relevance of nNOS in mitochondrial biogenesis is supported by our finding of increased sarcolemmal NADPHd activity in fibres with mitochondrial proliferation. Because sarcolemmal NADPHd activity was also increased in COX− fibres, we hypothesize that the increment in nNOS activity is an early event in COX deficiency that could result in a later increase in mitochondrial content (RRF). However, the sequence of events in the process of mitochondrial proliferation is not completely elucidated. Several studies demonstrated that NO donors are able to increase mitochondrial content in cultured myotubes, supporting that NO contributes, at least in part, to the process of mitochondrial biogenesis that ends up in the activation of PGC-1α^11^,^15^,^16^,^46^. The NOS isoform involved in mitochondrial proliferation may also vary depending on the cell type or conditions. For instance, in mouse brain, mitochondrial proliferation is induced through eNOS during normal conditions but during hypoxia the same process occurs via nNOS^47^. Recently, a study using differentiating myocytes demonstrated that nNOS is directed to the nucleus and promoted the S-nitrosylation of nuclear proteins to activate mitochondrial biogenesis^20^. Although a few points have been elucidated in recent years, the participation of NO in the mitochondrial biogenesis process is still poorly understood and is probably quite complex.

Our study did not demonstrate that Complex I or II defects have any effect on NADPHd activity. However, it is important to consider that these results should be viewed with caution because were based on results of only one patient. Other studies with larger number of patients and different genetic backgrounds are necessary to clarify other factors that could affect NOS activity in mitochondrial diseases. It is also worth mentioning that RRF are usually not found in muscle of patients with Complex I defects^44^, which supports our hypothesis that increased nNOS activity is related to induction of mitochondrial biogenesis.

Considering that stimulation of mitochondrial biogenesis is a possible therapeutic approach to mitochondrial diseases, it is important to know whether this induction could elicit adverse effects, such as apoptosis. Several studies showed contradictory results regarding apoptosis in muscle from patients with mitochondrial diseases^28^,^30^,^31^,^32^,^33^,^34^. Some studies found that apoptosis could be associated with muscle fibres with mitochondrial proliferation and high mutation load^28^,^30^; while others, studying similar groups of patients, did not confirm these findings^32^,^34^. Umaki *et al.* did not find increase in immunological markers such as Bax, p53, Fas and caspase 3, but found increase in DNA breaks and activation of deoxyribonuclease I, suggesting that another pathway could be involved in apoptosis in these samples. On the other hand, Ikezoe *et al.*^33^ found a small number of TUNEL-positive myonuclei in RRF and no significant increase of DNA fragmentation by electron microscopy. Likewise, we also found a very low frequency of apoptotic nuclei (up to 1.8%) when analyzing the entire muscle section of patients. Because our control group was small and the groups have heterogeneous number of patients, it was not possible to perform statistical analysis on the frequency of apoptotic nuclei in the different patient groups. Our control samples were limited due to the difficulty in finding normal diagnostic biopsies, but studies from other groups found similar results in control muscle samples, varying from 0 to less than 2% of fibres with apoptosis^28^,^30^,^34^. Although we have not studied apoptosis with immunological markers, the low proportion of apoptotic nuclei cannot be explained by a low sensitivity of the assay because other studies have demonstrated a good correlation between the TUNEL assay and immuno-markers in skeletal muscle of patients with mitochondrial diseases^28^,^30^. One possible reason to explain the low frequency of apoptotic nuclei in our study is the possibility of expression of an apoptotic inhibitor as proposed by Ikezoe *et al.*^33^. They found increased expression of the X-linked inhibitor of apoptosis protein in RRF and hypothesized that the cell may have mechanisms to stop the completion of the apoptosis^33^. Our results did not confirm any linkage of apoptotic nuclei with mitochondrial proliferation or mtDNA mutation. Regarding the patient with a Complex I defect due to a mutation in *MT-ND6*, our result was similar to Mirabella *et al.*^30^ who also found no apoptotic nuclei in the muscle from a patient with a mutation affecting *MT-ND4*, another subunit of Complex I^30^. The involvement of NO in the process of apoptosis is highly complex and sophisticated. NO has a dual function as it can either induce or inhibit apoptosis, depending on the concentration of the signal molecule^48^. Low concentrations of NO have anti-apoptotic actions while higher concentrations induce apoptosis^49^. The mechanisms involved in NO-dependent induction or inhibition of apoptosis are even more complicated considering that they are affected by several other factors such as reactive nitrogen species and substrate availability^49^. So based on our results, we speculate that the level of NO produced by sarcolemmal or sarcoplasmic NOS was not sufficient to induce apoptosis.

In summary, our results indicate that NOS activity, probably from the neuronal isoform, is increased in muscle fibres with mitochondrial proliferation, supporting the relevance nNOS in the mitochondrial biogenesis process. The NOS, located in the sarcoplasm, may be down regulated in COX deficient muscle fibres as consequence of NO involvement in the regulation of the respiratory chain. Apoptotic nuclei were found in a very small proportion of muscle fibres and were not associated with mitochondrial abnormalities or alterations in NOS activity. Taken together, our results show that NO can be considered a potential molecular target for pharmacological intervention to increase mitochondrial content and this approach may not be associated with increased apoptotic events.

## Methods

### Ethics Statement

Muscle biopsies were obtained for diagnostic purposes after written informed consent. The research project was approved by the Medical Research Ethics Committee at Universidade Federal de São Paulo and is in accordance with the Helsinki Declaration.

### Patients and muscle specimens

The study was performed on muscle specimens from 26 patients with mitochondrial myopathy, defined by the presence of mitochondrial abnormalities on muscle biopsy or presence of a pathogenic mtDNA mutation (Table 1). Patients’ samples did not present inflammatory cells infiltrates, adipose tissue infiltration or fibrosis. Fragments of the deltoid muscle were collected by open biopsy, immediately frozen in liquid nitrogen and stored at −80 °C. Control samples were selected among diagnostic muscle biopsies with no mitochondrial abnormalities, inflammatory infiltrate, neurogenic atrophy or type grouping. These patients had no clinical symptoms suggestive of any type of metabolic myopathy, positive family history for metabolic disease and no other ancillary test suggestive of mitochondrial disease. Muscle DNA screening did not show mtDNA deletions or frequent point mutations (m.8344A > G, m.3243A > G, m.8993T > G).

### Histochemistry

In order to allow the evaluation of the same muscle fibre in different histochemical assays, 10 μm serial muscle sections were obtained from each muscle sample. Individual muscle fibres were used to evaluate fibre type (adenosine triphosphatase, ATPase, pH 9.4 assay), mitochondrial enzyme activities (COX and SDH), NOS activity (nicotinamide adenine dinucleotide phosphate diaphorase histochemistry, NADPHd) and presence of apoptotic nuclei (TUNEL).

Histochemical stainings of SDH, COX and ATPase pH 9.4 were performed as previously described^25^,^50^. SDH and COX histochemistry were performed at 37 °C for 60 min. For SDH staining, samples were incubated in 5 mM phosphate buffer, pH 7.6, containing 5 mM ethylene diamine tetraacetic acid (EDTA), 1 mM potassium cyanide, 0.2 mM phenazine methosulfate, 50 mM succinic acid, 1.5 mM nitro blue tetrazolium. For COX staining, samples were incubated in 5 mM phosphate buffer, pH 7.4, containing 0.1% diaminobenzidine, 0.1% cytochrome c (from horse heart), 0.02% catalase. For ATPase pH 9.4 histochemistry, muscle sections were pre-incubated with 2% calcium chloride in 0.1 M sodium barbital buffer, pH 9.4 at room temperature for 15 min. Followed by incubation in the same buffer at pH 9.4 with adenosine-5-triphosphate (1.25 mg/ml) for 30 min at 37 °C and later in 2% cobalt chloride for 7 min at room temperature. Then the sections were incubated with 1% ammonium sulfate, washed with water and dehydrated with ethanol and xylene. NADPHd histochemistry was performed as previously described by Frandsen *et al.*^43^. Briefly, muscle sections were fixed in 2% formaldehyde for 10 min and rinsed with phosphate buffered saline (PBS). Then the sections were incubated in PBS, pH 7.4, containing nitroblue tetrazolium (0.1 mg/ml), β-NADPH (1 mg/ml) and 0.3% Triton X-100 for 60 min at 37 °C. Specificity of NADPHd histochemistry as a marker of NOS activity was verified by immunohistochemistry using antibodies against nNOS, eNOS and iNOS, from BIOMOL Enzo Life Sciences (Supplementary Fig. S3).

### Quantitative analysis of histochemical reactions

Quantification of histochemical SDH, COX and NADPHd activities was performed using the public domain NIH Image J 1.38x Software, as previously described^4^,^51^ with some modifications. Stained muscle sections were captured using a digital camera, with 20x objective lens, at constant light intensity, condenser setting and exposure time. The images were saved as TIFF (8 bit) format and calibrated with an optical density (OD) scale. SDH, COX and sarcoplasmic NADPHd stainings were quantified by measuring the mean O.D. of the circumscribed area of the sarcoplasm of each muscle fibre with an interactive cursor (polygon selection; Fig. 1). Sarcolemmal NADPHd intensity was obtained with the mean O.D. inside the squares (10 × 10 pixels) placed at 12 sites (three per quarter) on the sarcolemma of each fibre. To avoid saturated staining, the ideal incubation time of 60 min was established based on results obtained between 15 and 90 min incubation (Supplementary Fig. S4).

In all patients’ slides, the fibres were classified as normal or with mitochondrial abnormalities. Fibres were considered normal when SDH and COX stainings were within the normal O.D. range, which was obtained after quantification of 248 fibres (type I = 133; type II = 115) of six control muscle biopsies. In each patient’ slide, the mean O.D. of normal fibres was considered as 100% and the values were expressed using this mean as reference to allow the comparison among all patient samples. As shown in Table 2, abnormal muscle fibres were classified as RRF/COX+ (with mitochondrial proliferation and COX activity similar to normal fibres), RRF/COXdef (with mitochondrial proliferation and disproportional low COX activity), RRF/COX− (with mitochondrial proliferation and low COX activity) or COX− (with low COX activity but no mitochondrial proliferation).

### TUNEL assay

Apoptotic nuclei were detected in serial 5 μm thick muscle sections by the TUNEL assay (TdT-FragEL™ DNA Fragmentation Detection Kit, Calbiochem) according to manufacturer instructions (Supplementary Fig. S5). Positive control for muscle fibres was obtained by detecting apoptotic nuclei in muscle sections treated with deoxyribonuclease (RQ1 RNase-Free DNase, Promega) for 20 min at 37 °C. Negative controls were generated treating specimens with Tris-buffered saline, instead of terminal deoxynucleotidyl transferase enzyme in the reaction step.

### Statistical analyses

Statistical analyses were performed using Prism 6 for MacOS X (Graph Pad Software Inc., La Jolla California USA). Parametric or non-parametric tests were applied considering the Shapiro-Wilk normality test. Statistical significance was established at p < 0.05.

## Additional Information

**How to cite this article**: Rodrigues, G. S. *et al.* Integrated analysis of the involvement of nitric oxide synthesis in mitochondrial proliferation, mitochondrial deficiency and apoptosis in skeletal muscle fibres. *Sci. Rep.* **6**, 20780; doi: 10.1038/srep20780 (2016).

## Supplementary Material

Supplementary Information

## Figures and Tables

**Table 1:** Clinical features, genetic etiology, percentage of RRF and COX deficient fibres.

pat.	g	age (y)	phenotype	duration (y)	genetic etiology (gene)	RRF (%)	COX def. (%)
1*	M	12	mental retard.	ND	control	0	0
2*	M	3	mental retard.	ND	control	0	0
3*	M	7	myocl. epilep.	ND	control	0	0
4*	F	7	myocl. epilep.	ND	control	0	0
5*	F	32	fibromyalgia	12	control	0	0
6	M	69	CPEO	4	mtDNA mult.del. (multiple)	5	9
7	F	45	CPEO	15	mtDNA mult.del. (multiple)	2	3
8	F	45	CPEO	13	mtDNA mult.del. (multiple)	9	10
9	F	53	CPEO	26	mtDNA mult.del. (multiple)	18	20
10	M	64	CPEO	1	mtDNA mult.del. (multiple)	9	12
11	F	38	MNGIE like	19	mtDNA mult.del. (multiple)	6	4
12	F	63	CPEO	2	mtDNA mult.del. (multiple)	6	10
13	M	33	MNGIE like	9	mtDNA mult.del. (multiple)	4	13
14	F	38	CPEO	18	mtDNA sing. del. (multiple)	8	6
15	M	32	CPEO	ND	mtDNA sing. del. (multiple)	11	15
16	F	40	CPEO	15	mtDNA sing. del. (multiple)	5	12
17	M	16	CPEO	3	mtDNA sing. del. (multiple)	29	61
18	F	11	CPEO	3	mtDNA sing. del. (multiple)	4	7
19	F	18	CPEO	11	mtDNA sing. del. (multiple)	6	18
20	M	24	CPEO	ND	mtDNA sing. del. (multiple)	2	2
21	M	38	CPEO	4	mtDNA sing. del. (multiple)	5	6
22	M	16	CPEO	4	mtDNA sing. del. (multiple)	3	25
23	F	27	CPEO	ND	mtDNA sing. del. (multiple)	33	36
24	F	32	CPEO	ND	mtDNA sing. del. (multiple)	3	5
25	M	4	MELAS	2	m.3243A>G (*MT-TL1*)	12	1
26	M	45	exerc. intol.	1	m.3243A>G (*MT-TL1*)	18	17
27	M	19	MELAS	4	m.3243A>G(*MT-TL1*)	17	6
28	F	34	CPEO	22	m.3251A>G(*MT-TL1*)	26	23
29	M	10	CPEO	5	m.4369_4370insA(*MT-TQ*)	19	89
30	F	28	myopathy	21	non-identified nuclear gene	40	27
31	M	26	dystonia	6	m.14459G>A (*MT-ND6*)	0	0

* = patients used as controls for the TUNEL assay. pat = patient; g = gender; age = age at biopsy; M = male; F = female; y = years; MNGIE = mitochondrial neurogastrointestinal encephalopathy; CPEO = chronic progressive external ophthalmoplegia; retard. = retardation; myocl. epilep. = myoclonic epilepsy; CPEO = chronic progressive external ophthalmoplegia; MELAS = mitochondrial encephalomyopathy with lactic acidosis and stroke like episodes; exerc. intol. = exercise intolerance; duration = duration of disease; ND = not defined, mtDNA = mitochondrial DNA; mult. del. = multiple deletions; sing. del = single deletion; nDNA = nuclear DNA; RRF = ragged red fibres; COX def. = cytochrome-c-oxidase deficient fibres.

**Table 2:** Classification of muscle fibres with mitochondrial alterations according to histochemical quantification.

fibre type	mitochondrial alteration	SDH activity (%)	COX activity (%)	SDH/COX	N
I	normal	56.9–143.9	53.1–161.1	0.6–1.5	477
	RRF/COX+	>143.9	>53.1	0.6–1.5	48
	RRF/COXdef	>143.9	53.1-161.1	>1.5	119
	RRF/COX−	>143.9	<53.1		322
	COX−	56.9–143.9	<53.1		168
II	Normal	55.1–161.1	47.5–152.9	0.6–1.7	365
	RRF/COX+	>161.1	>47.5	0.6–1.7	49
	RRF/COXdef	>161.1	47.5–152.9	>1.7	56
	RRF/COX−	>161.1	<47.5		150
	COX−	55.1–161.1	<47.5		223

RRF: ragged red fibres; COX+: normal COX activity; COXdef: partial COX deficiency; COX−: total COX deficiency; N: number of fibres.

**Table 3:** Mitochondrial abnormalities and NADPHd activity in fibres with and without apoptotic nuclei.

	mitoc. abnormality			SP-NADPHd				SL-NADPHd		
	abnl	nl	total	↑	nl	↓	total	↑	nl	total
	(%)	(%)	(%)	(%)	(%)	(%)	(%)	(%)	(%)	(%)
TUNEL +	14	24	38	6	30	2	38	6	32	38
	(36.8)	(63.2)	(100)	(15.8)	(78.9)	(5.3)	(100)	(15.8)	(84.2)	(100)
TUNEL −	712	936	1648	94	675	60	829	152	677	829
	(43.2)	(56.8)	(100)	(11.3)	(81.4)	(7.2)	(100)	(18.3)	(81.7)	(100)

mitoc.: mitochondrial; abnl: abnormal; nl: normal; SP-NADPHd: sarcoplasmic-NADPH diaphorase; SL-NADPHd: sarcolemmal NADPH diaphorase; ↑: increased; ↓: decreased; +: positive; -: negative.
