# Corrigendum: Integrated analysis of the involvement of nitric oxide synthesis in mitochondrial proliferation, mitochondrial deficiency and apoptosis in skeletal muscle fibres

**DOI:** 10.1038/srep30099

**Published:** 2016-07-20

**Authors:** Gabriela Silva Rodrigues, Rosely Oliveira Godinho, Beatriz Hitomi Kiyomoto, Juliana Gamba, Acary Souza Bulle Oliveira, Beny Schmidt, Célia Harumi Tengan

Scientific Reports 6: Article number: 2078010.1038/srep20780; published online: 02092016; updated: 07202016

The Acknowledgements section in this Article is incomplete.

“We are grateful to Prof. Alberto Alain Gabbai and Dr. Wladimir B. V. R. Pinto for valuable suggestions on the manuscript. This study was supported by research grants 2007/03145-9 (CHT), 2007/00808-7 (GSR), São Paulo Research Foundation (FAPESP) and Coordenação de Aperfeiçoamento de Pessoal de Ensino Superior (CAPES) to GSR and JG.”

should read:

“We are grateful to Prof. Alberto Alain Gabbai and Dr. Wladimir B. V. R. Pinto for valuable suggestions on the manuscript. This study was supported by research grants 2007/03145-9 (CHT), 2007/00808-7 (GSR), 2016/03920-1 (CHT), São Paulo Research Foundation (FAPESP) and Coordenação de Aperfeiçoamento de Pessoal de Ensino Superior (CAPES) to GSR and JG”.

