# Supplementary material for: Integrated analysis of the involvement of nitric oxide synthesis in mitochondrial proliferation, mitochondrial deficiency and apoptosis in skeletal muscle fibres
Source: Sci Rep. 2016 Feb 9;6:20780. doi: 10.1038/srep20780 (PMC4746761; doi:10.1038/srep20780)
Supplement: Supplementary Information [file srep20780-s1.pdf]

Supplementary Information

Integrated analysis of the involvement of nitric oxide synthesis in mitochondrial proliferation, mitochondrial deficiency and apoptosis in skeletal muscle fibres

Gabriela Silva Rodrigues[1], Rosely Oliveira Godinho[2], Beatriz Hitomi Kiyomoto[1], Juliana Gamba[1], Acary Souza Bulle Oliveira[1], Beny Schmidt[3], Célia Harumi Tengan[1]*

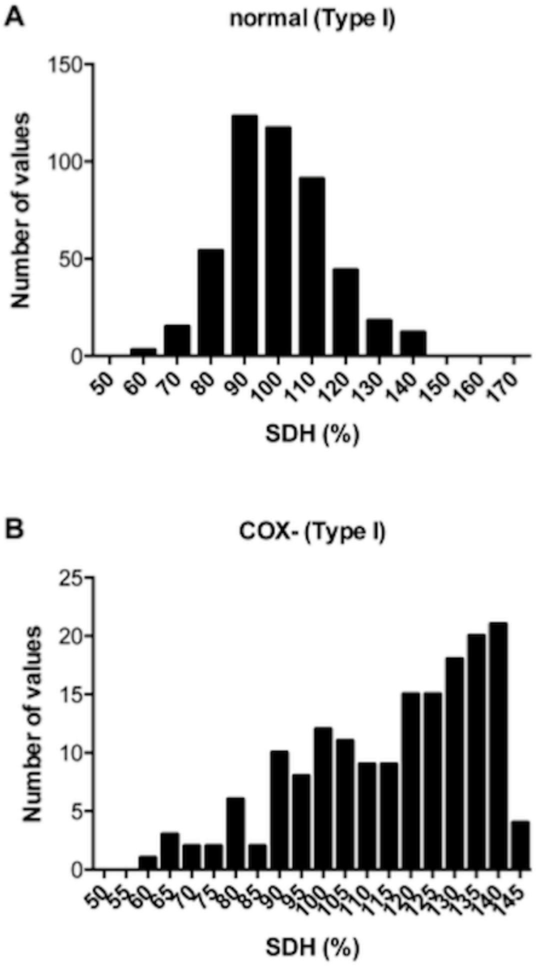

Supplementary Figure S1. Frequency distribution of SDH staining in type I normal and COX-fibres.

Frequency distribution of results obtained in the quantification of SDH staining in type I normal (A) and COX- (B) fibres. The distribution of COX- fibres is within the range of normal fibres, but there is a predominance of higher values, with a median of 119.5% while the median of normal fibres is 97.7%.

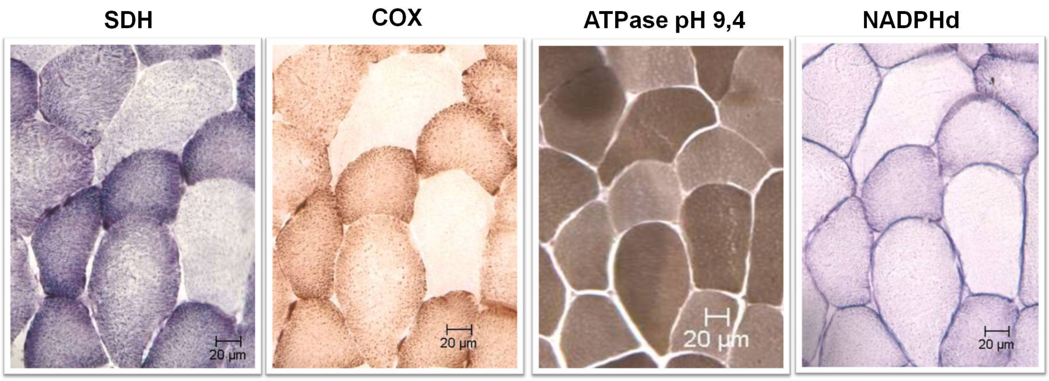

Supplementary Figure S2. Histochemical stainings in muscle sections from a patient with a Complex I defect.

Serial sections from a muscle biopsy of a patient with a Complex I defect are shown. SDH and COX histochemical stainings show a normal pattern with no mitochondrial proliferation and no COX deficiency. ATPase (pH 9.4) demonstrate the distribution of type I (light) and II (dark) fibres. NADPH diaphorase histochemistry also show a normal pattern with staining in the sarcolemma and lighter staining in the sarcoplasm of type II fibres.

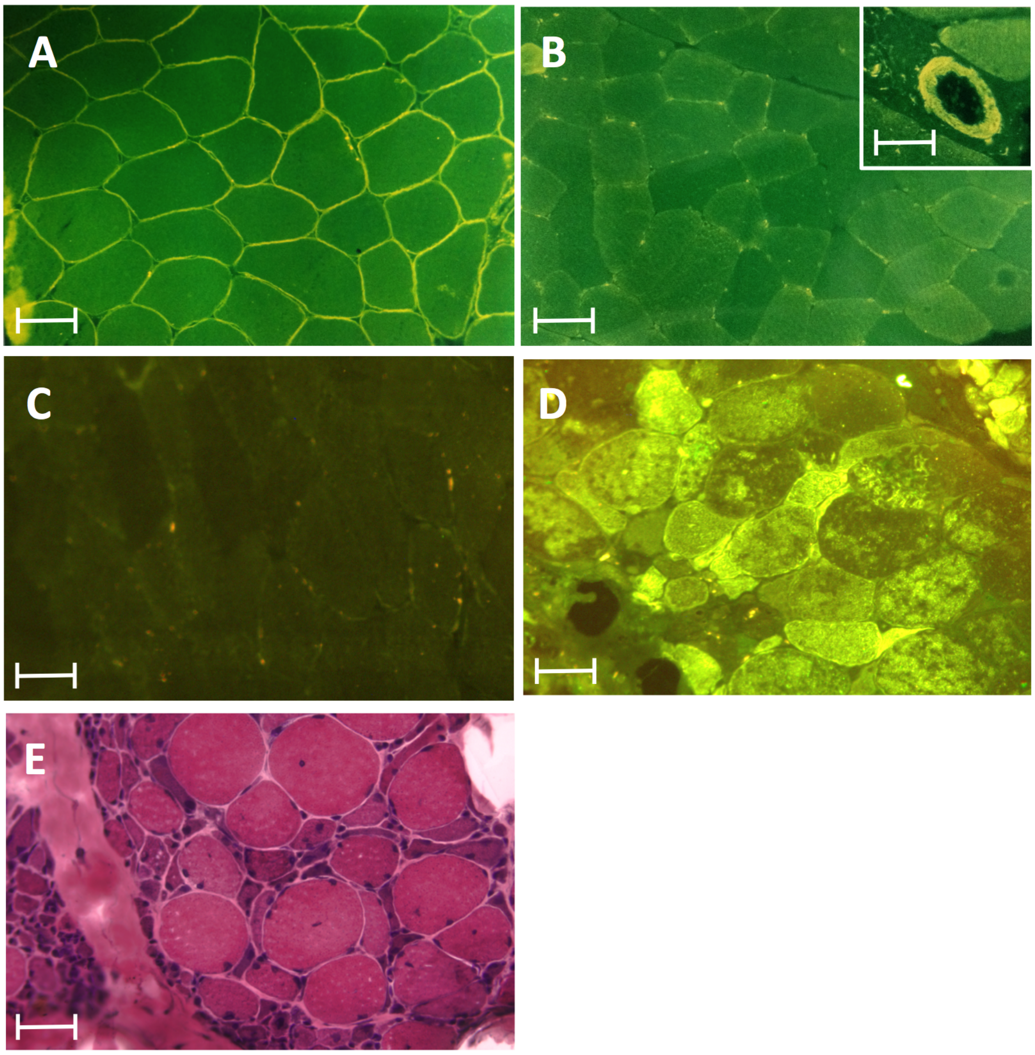

Supplementary Figure S3. Expression of NOS isoforms in skeletal muscle.

Immunohistochemistry of normal skeletal muscle shows that nNOS is expressed in the sarcolemmal membrane (A), while eNOS is detected in the sarcoplasm (B) and muscle vessel (inset). Incubation with an antibody against the inducible isoform (iNOS) shows that iNOS is not expressed in muscle with mitochondrial abnormalities (C). Intense reactivity to the iNOS antibody is demonstrated in muscle sample with inflammatory features (D), showing that this antibody is able to detect iNOS expression in muscle. Haematoxylin and eosin staining (E) shows inflammatory features found in this sample, such as macrophagy, inflammatory infiltrate, necrosis, atrophic fibres with fibrosis. Bar=50μm.

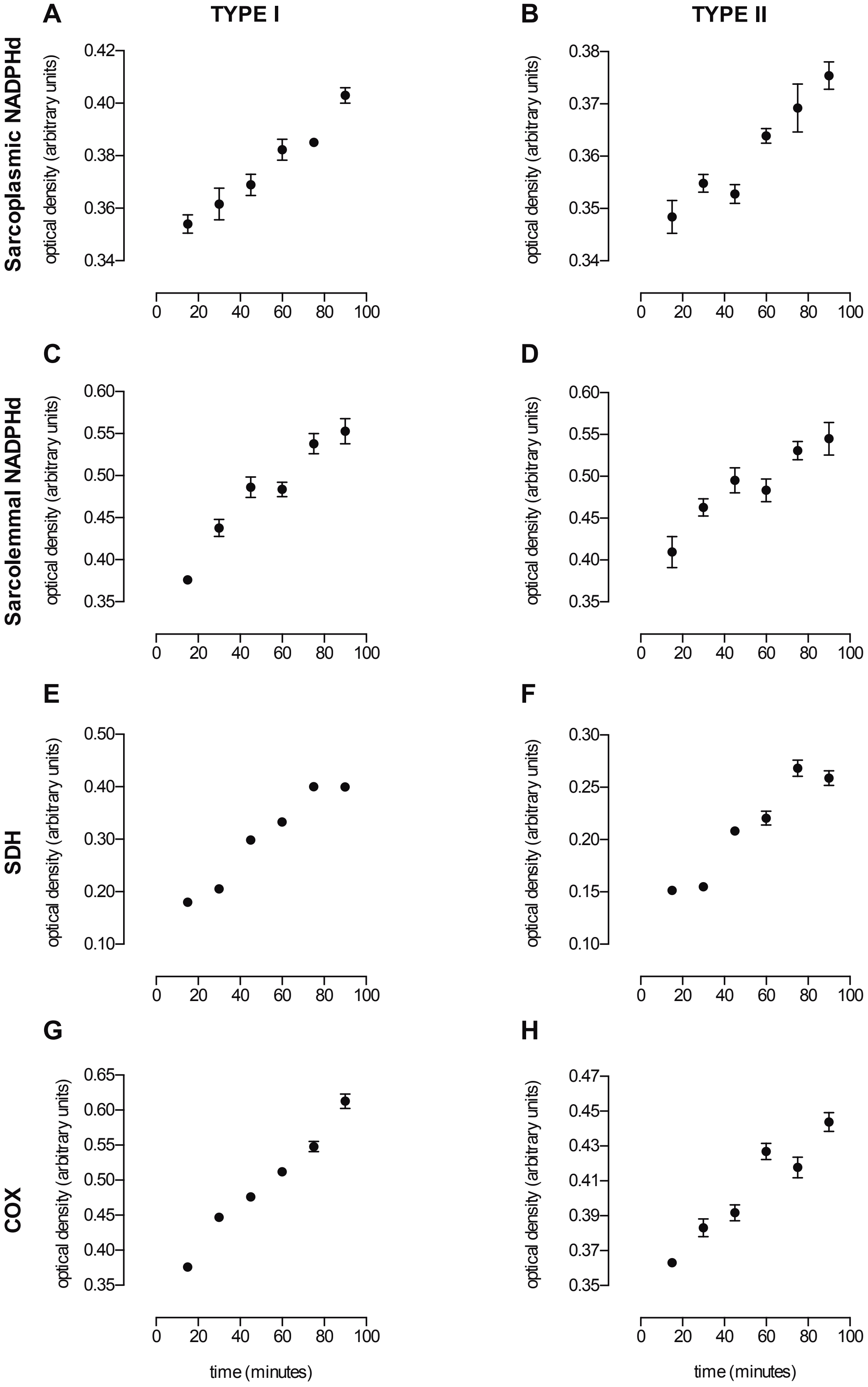
A
TYPE I
B
TYPE II
Sarcoplasmic NADPHd
Sarcolemmal NADPHd
SDH
COX
C
D
E
F
G
H
optical density (arbitrary units)
time (minutes)
0.42
0.40
0.38
0.36
0.34
0.37
0.35
0.60
0.55
0.50
0.45
0.40
0.35
0.30
0.25
0.20
0.15
0.10
0.65
0.47
0.45
0.43
0.41
0.39
0.37
0
20
40
60
80
100

Supplementary Figure S4. Linearity of histochemical stainings.

Histochemical reactions were performed with 10μm muscle sections in 15, 30, 45, 60, 75 and 90 min. Measurements of sarcoplasmic (A, B) and sarcolemmal (C, D) NADPHd, SDH (E, F), COX (G, H) stainings were obtained in type I and II muscle fibres and plotted against time. We show that results obtained with a 60 min incubation time, as we used in the experiments, were within the linear phase of all histochemical reactions (A-H). Dots are means with standard errors. N=10 fibres for each time point.

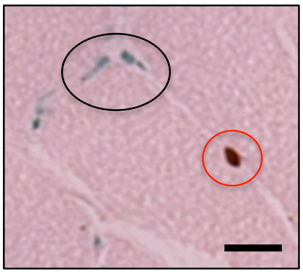

Supplementary Figure S5. Detection of apoptotic nuclei in skeletal muscle.

The TUNEL assay shows brown apoptotic nuclei (red open circle) and green stained normal nuclei (black open circle) in skeletal muscle fibres. Scale bar= 20μm.
